# Optimizing the treatment of BRAF mutant melanoma

**DOI:** 10.1186/gm547

**Published:** 2014-04-28

**Authors:** Jeff Settleman

**Affiliations:** 1 Discovery Oncology, Genentech, 1 DNA Way, South San Francisco, CA 94080, USA

## Abstract

Selective inhibitors of the kinases BRAF and MEK for the treatment of patients with otherwise refractory *BRAF* mutant melanoma have demonstrated impressive efficacy, and combination treatment with these agents may prove to be even more effective. However, these drugs are not curative, mainly because of the relatively rapid development of drug resistance. Furthermore, they can produce undesired, and even unanticipated, side effects, including the emergence of neoplastic lesions harboring activating *RAS* mutations. Two recent reports reveal new considerations for the optimal approach to targeting this key oncogenic pathway in melanoma, highlighting the importance of combination treatment and therapeutic scheduling.

## Challenges in targeting the BRAF pathway

The discovery of a highly recurrent mutant allele of the gene encoding the RAF-family kinase BRAF in human melanoma through cancer genome resequencing has set the stage for revolutionary new treatment strategies for this challenging disease [1]. This BRAF mutation, V600E, which has been detected in 50 to 60% of human melanomas, results in activation of the kinase; this drives the downstream MEK-ERK kinase signaling cascade, leading to proliferation and survival of cancer cells. Selective targeting of the BRAF or MEK kinases using targeted small-molecule inhibitory drugs in patients with BRAF V600E mutant melanoma has been associated with impressive clinical responses in more than half of treated patients - despite the presence of systemic disease [2,3]. However, as with other kinase-targeted anti-cancer drugs, the clinical benefit is typically short-lived due to the emergence of drug resistance, through a variety of molecular mechanisms [4]. Such findings have prompted substantial efforts to elucidate these mechanisms and to use that information to guide the discovery of second-generation drugs, drug combination strategies, and novel treatment dosing and scheduling regimens. The goal is to overcome or prevent drug resistance while minimizing treatment-associated toxicity.

Another challenge to the use of these new agents - particularly for the BRAF inhibitors - is the unanticipated, paradoxical activation of ERK signaling in patients' cells that do not harbor the *BRAF* mutation [5]. Thus, in cells with wild-type *BRAF* alleles, the current BRAF inhibitors in fact promote ERK signaling through an unintended allosteric activation of dimers of BRAF with the related kinase CRAF, leading to MEK and ERK activation. This appears to be particularly relevant in cells harboring an activated allele of *NRAS*, which encodes a RAS-family kinase. Indeed, approximately 20% of melanoma patients treated with the BRAF inhibitors vemurafenib or dabrafenib develop keratoacanthomas and/or squamous cell carcinomas of the skin that can harbor activating *RAS* mutations (mostly *HRAS*), suggesting that the oncogenic potential of these *RAS* mutations is realized specifically in the context of drug treatment [6]. More recent clinical evaluation of BRAF and MEK inhibitor combination treatment in melanoma suggests that this regimen reduces the incidence of these skin lesions, presumably as a result of the ability of the MEK inhibitor to suppress the activation of ERK downstream of RAS-driven BRAF:CRAF-dependent MEK activity [7].

Despite the dramatic clinical benefit associated with these new 'personalized' drug treatments for melanoma patients, the issues described above highlight some important remaining challenges, and the many published reports on this topic over the past 3 years point to the intense interest in better understanding this key pathway in melanoma. Here, I highlight two such recent studies [8,9] that address the treatment of *BRAF* mutant melanoma, revealing new therapeutic strategies that can potentially improve outcomes for such patients.

## Managing NRAS-mutant leukemia through intermittent co-treatment with BRAF and MEK inhibitors

The first of these reports, from Paul Chapman and colleagues [8], describes a case study of a melanoma patient with a *BRAF* mutant tumor who was diagnosed with chronic myelomonocytic leukemia (CMML) while undergoing treatment with vemurafenib. The leukemic cells in this patient were associated with an activating NRAS G12R mutation, consistent with a potential role for vemurafenib in inducing ERK activation, similar to that seen in the *RAS*-mutant skin lesions observed in melanoma patients treated with BRAF inhibitors. Thus, vemurafenib treatment may have accelerated the development of a previously unsuspected RAS-mutant leukemia. The patient's melanoma was effectively managed with intermittent vemurafenib treatment, and the authors [8] observed that combining BRAF and MEK inhibitors effectively reduced the proliferation of CMML cells. Although CMML-derived DNA revealed that *NRAS*-mutant leukemic cells were still detectable following treatment, the patient had experienced symptomatic improvement over an 85-week treatment period, until the time of the report.

The investigators [8] also examined leukemia cell line models with or without an *NRAS* mutation to demonstrate that BRAF/MEK co-treatment could inhibit proliferation of *NRAS* mutant leukemic cells, and that, unlike BRAF inhibition alone, which promoted ERK activation, co-treatment resulted in effective suppression of ERK activation. They concluded that the use of intermittent dosing with combined MEK and BRAF inhibitors prevented the paradoxical ERK activation normally seen with sustained BRAF inhibition in cells that do not harbor an activating *BRAF* mutation. The authors noted that the potential anti-leukemic effects of MEK inhibitor treatment may have been partially mitigated by co-treatment with vemurafenib, which promotes ERK activation in the *NRAS* mutant leukemic cells. Although this study reflects the experience of a single patient, MEK inhibition appears to be effective in *RAS*-mutant myeloid malignancies, as evidenced by some early clinical studies [10], and the authors [8] suggest that intermittent treatment in this context may reduce associated drug toxicities while yielding enhanced pathway inhibition.

## Epidermal growth factor receptor expression in BRAF mutant melanoma

In a second report, from Rene Bernards and colleagues [9], a novel mechanism of resistance to BRAF inhibition in melanoma was described. Several molecular mechanisms associated with acquired and intrinsic resistance to BRAF inhibition have now been reported, including activating mutations in *NRAS* and *MEK1*, amplification or alternative splicing of *BRAF* itself, and increased expression of the COT1 kinase or various receptor tyrosine kinases [4]. Building on their previous observation of intrinsic resistance of *BRAF* mutant colorectal cancers to epidermal growth factor receptor (EGFR) activation [11], these investigators [9] explored a potential role for EGFR in acquired resistance to BRAF inhibition in *BRAF* mutant melanoma. Indeed, they found that post-treatment melanoma biopsies revealed evidence of increased EGFR expression.

Then, to determine how EGFR expression is regulated in melanocytes, a cell type that does not normally express EGFR, Bernards and colleagues [9] conducted an RNA interference screen using a library of short hairpin RNAs designed to target each of the more than 600 genes encoding various chromatin-associated regulatory proteins, including several histone modifying enzymes. That screen identified the SRY (sex determining region Y)-box 10 (*SOX10*) gene as a regulator of EGFR expression in four different tested melanoma cell lines. Moreover, *SOX10* knockdown was sufficient to confer vemurafenib resistance, and this was associated with increased EGFR expression. Additional studies revealed that SOX10-mediated suppression of transforming growth factor β receptor expression was responsible for SOX10's repressive effect on expression of EGFR as well as on other receptor tyrosine kinases that appear to contribute to vemurafenib resistance. The role of SOX10 in regulating EGFR in melanoma was further supported by the discovery of an inverse relationship between *SOX10* and *EGFR* gene expression in a panel of *BRAF* mutant melanoma cell lines, as well as in a few *BRAF* mutant melanoma patient samples following treatment-associated resistance [9].

Interestingly, these investigators [9] had also observed that EGFR expression in *BRAF* mutant melanoma cells was surprisingly detrimental to their proliferative capacity, and was only tolerated in the context of BRAF inhibition. This finding suggests that, in the absence of drug, adaptive mechanisms underlying drug resistance may come at a 'fitness cost', potentially explaining clinical findings of reversible drug resistance in vemurafenib-treated melanoma patients. Thus, in patients whose cancer progresses on treatment and are consequently taken off therapy, residual tumor cells that lack the proliferation-compromising resistance mechanism (EGFR expression in this case) may have a selective growth advantage, leading to their enrichment as a dominant clone within the relapsed tumor. In such a patient, re-treatment with a BRAF inhibitor would be expected to lead to partial tumor regression. This highlights the dynamic nature of tumor cell heterogeneity in the context of treatment sensitivity and resistance, and the potentially important role of treatment scheduling in producing optimal clinical benefit.

In summary, these two recent reports [8,9] provide important new insights into some of the complexities associated with targeting the BRAF-MEK-ERK signaling pathway in *BRAF* mutant melanoma. Although they address distinct aspects of treatment-associated challenges (acquired resistance and oncogenic side effects), they both relate to the less appreciated issue of treatment scheduling. This will undoubtedly become a more prominent topic of study as both laboratory-based and clinical investigators continue to optimize strategies for combination treatments that can manage these tumors more effectively and safely.

## Abbreviations

CMML: Chronic myelomonocytic leukemia; EGFR: Epidermal growth factor receptor; SOX: Sex determining region Y-box.

## Competing interests

The author is an employee of Genentech, Inc. and a shareholder of Roche Pharmaceuticals.
